# Supplementary material for: Differences in and associations between belief in just deserts and human rights restrictions over a 3-year period in five countries during the COVID-19 pandemic
Source: PeerJ. 2023 Sep 28;11:e16147. doi: 10.7717/peerj.16147 (PMC10542388; doi:10.7717/peerj.16147)
Supplement: Supplemental Information 2 [file peerj-11-16147-s002.docx]

Table S1. Spearman-Brown coefficient for belief in just deserts by country and year.

| Year | Japan | The United States | The United Kingdom | Italy | China |
| --- | --- | --- | --- | --- | --- |
| 2020 | 0.897 | 0.747 | 0.797 | 0.651 | 0.565 |
| 2021 | 0.908 | 0.833 | 0.857 | 0.664 | 0.669 |
| 2022 | 0.898 | 0.806 | 0.835 | 0.731 | 0.767 |
