# Supplementary material for: Differences in and associations between belief in just deserts and human rights restrictions over a 3-year period in five countries during the COVID-19 pandemic
Source: PeerJ. 2023 Sep 28;11:e16147. doi: 10.7717/peerj.16147 (PMC10542388; doi:10.7717/peerj.16147)
Supplement: Supplemental Information 3 [file peerj-11-16147-s003.docx]

Table S2. Cronbach’s α for human rights restriction by country and year.

| Year | Japan | The United States | The United Kingdom | Italy | China |
| --- | --- | --- | --- | --- | --- |
| 2020 | 0.718 | 0.743 | 0.758 | 0.726 | 0.814 |
| 2021 | 0.754 | 0.806 | 0.823 | 0.682 | 0.836 |
| 2022 | 0.777 | 0.818 | 0.818 | 0.716 | 0.844 |
