# Supplementary material for: Differences in and associations between belief in just deserts and human rights restrictions over a 3-year period in five countries during the COVID-19 pandemic
Source: PeerJ. 2023 Sep 28;11:e16147. doi: 10.7717/peerj.16147 (PMC10542388; doi:10.7717/peerj.16147)
Supplement: Supplemental Information 4 — Simple main effects are adjusted by Bonferroni correction: P values are multiplied by the number of groups (i.e., 5 for countries and 3 for years). BJD is adjusted for different covariates: age (33.2), gender (women = 0.60), academic career (university degree or higher = 0.64), children under junior high school age in the family (presence = 0.28), and elderly people over 65 in the family (presence = 0.22). Interaction: P = 0.002, partial η2 = 0.004. [file peerj-11-16147-s004.docx]

Table S3. Belief in just deserts (BJD) by country and year. Data are shown as the mean (95% confidence interval). Simple main effects are adjusted by Bonferroni correction: *P* values are multiplied by the number of groups (i.e., 5 for countries and 3 for years). BJD is adjusted for different covariates: age (33.2), gender (women = 0.60), academic career (university degree or higher = 0.64), children under junior high school age in the family (presence = 0.28), and elderly people over 65 in the family (presence = 0.22). Interaction: *P* = 0.002, partial η^2^ = 0.004.

|  | Japan | The United States | The United Kingdom | Italy | China |
| --- | --- | --- | --- | --- | --- |
| 2020 | 2.30 (2.22–2.39)^a; Y^ | 1.50 (1.42–1.59)^b, c; Y^ | 1.44 (1.36–1.53)^c; Y^ | 1.64 (1.57–1.72)^b; Y^ | 1.67 (1.59–1.75)^b; Y^ |
| 2021 | 2.52 (2.44–2.61)^a; X^ | 1.94 (1.86–2.03)^b; X^ | 1.66 (1.58–1.75)^d; X^ | 1.87 (1.78–1.95)^b, c; X^ | 1.74 (1.65–1.82)^c, d; X, Y^ |
| 2022 | 2.54 (2.45–2.63)^a; X^ | 1.86 (1.77–1.94)^b; X^ | 1.62 (1.53–1.71)^c; X^ | 1.75 (1.67–1.84)^b, c; X, Y^ | 1.81 (1.72–1.89)^b; X^ |

a-d: Different letters represent significant differences (*P* < 0.05) among countries as a simple main effect.

X-Y: Different letters represent significant differences (*P* < 0.05) between years as a simple main effect.
