# Supplementary material for: Differences in and associations between belief in just deserts and human rights restrictions over a 3-year period in five countries during the COVID-19 pandemic
Source: PeerJ. 2023 Sep 28;11:e16147. doi: 10.7717/peerj.16147 (PMC10542388; doi:10.7717/peerj.16147)
Supplement: Supplemental Information 5 — Data are shown as the mean (95% confidence interval). Simple main effects are adjusted by Bonferroni correction: P values are multiplied by the number of groups (i.e., 5 for countries and 3 for years). HRR is adjusted for different covariates: age (33.2), gender (women = 0.60), academic career (university degree or higher = 0.64), children under junior high school age in the family (presence = 0.28), and elderly people over 65 in the family (presence = 0.22). Interaction: P < 0.001, partial η2 = 0.023. [file peerj-11-16147-s005.docx]

Table S4. Human rights restriction (HRR) by country and year. Data are shown as the mean (95% confidence interval). Simple main effects are adjusted by Bonferroni correction: *P* values are multiplied by the number of groups (i.e., 5 for countries and 3 for years). HRR is adjusted for different covariates: age (33.2), gender (women = 0.60), academic career (university degree or higher = 0.64), children under junior high school age in the family (presence = 0.28), and elderly people over 65 in the family (presence = 0.22). Interaction: *P* < 0.001, partial η^2^ = 0.023.

|  | Japan | The United States | The United Kingdom | Italy | China |
| --- | --- | --- | --- | --- | --- |
| 2020 | 3.68 (3.58–3.78)^e; X^ | 4.06 (3.96–4.16)^d; X^ | 4.93 (4.83–5.02)^b; X^ | 4.47 (4.38–4.56)^c; X^ | 6.01 (5.91–6.10)^a; X^ |
| 2021 | 3.49 (3.39–3.59)^c; X, Y^ | 3.87 (3.77–3.97)^b; Y^ | 4.03 (3.93–4.13) | 4.04 (3.94–4.14)^b; Y^ | 5.88 (5.77–5.98)^a; X^ |
| 2022 | 3.33 (3.22–3.43)^c; Y^ | 3.51 (3.41–3.61)^c; Z^ | 3.79 (3.69–3.90)^b; Z^ | 3.79 (3.69–3.89)^b; Z^ | 5.88 (5.78–5.98)^a; X^ |

a-e: Different letters represent significant differences (*P* < 0.05) among countries as a simple main effect.

X-Z: Different letters represent a significant difference (*P* < 0.05) among years as a simple main effect.
