# Supplementary material for: Differences in and associations between belief in just deserts and human rights restrictions over a 3-year period in five countries during the COVID-19 pandemic
Source: PeerJ. 2023 Sep 28;11:e16147. doi: 10.7717/peerj.16147 (PMC10542388; doi:10.7717/peerj.16147)
Supplement: Supplemental Information 6 — Data are shown as the mean (95% confidence interval). Simple main effects are adjusted by Bonferroni correction: P values are multiplied by the number of groups (i.e., 5 for countries and 3 for years). Interaction: P ¡ 0.001, partial η2 = 0.010. [file peerj-11-16147-s006.docx]

Table S5. Belief in just deserts by country and year only for the first-time participants. Data are shown as the mean (95% confidence interval). Simple main effects are adjusted by Bonferroni correction: *P* values are multiplied by the number of groups (i.e., 5 for countries and 3 for years). Interaction: *P* < 0.001, partial η^2^ = 0.010.

|  | Japan | The United States | The United Kingdom | Italy | China |
| --- | --- | --- | --- | --- | --- |
| 2020 | 2.27 (2.19–2.36)^a; Y^ | 1.50 (1.41–1.58)^c; Y^ | 1.41 (1.33–1.50)^c; X^ | 1.67 (1.59–1.74)^b; Y^ | 1.77 (1.70–1.85)^b; X^ |
| 2021 | 2.53 (2.42–2.64)^a; X^ | 1.94 (1.83–2.05)^b; X^ | 1.60 (1.47–1.73)^c; X^ | 1.94 (1.84–2.03)^b; X^ | 1.74 (1.65–1.82)^c; X^ |
| 2022 | 2.73 (2.59–2.87)^a; X^ | 1.82 (1.72–1.92)^b; X^ | 1.49 (1.37–1.62)^c; X^ | 1.77 (1.63–1.90)^b; X, Y^ | 1.86 (1.77–1.94)^b; X^ |

a-c: Different letters represent significant differences (*P* < 0.05) among countries as a simple main effect.

X-Y: Different letters represent significant differences (*P* < 0.05) between years as a simple main effect.
