# Supplementary material for: Differences in and associations between belief in just deserts and human rights restrictions over a 3-year period in five countries during the COVID-19 pandemic
Source: PeerJ. 2023 Sep 28;11:e16147. doi: 10.7717/peerj.16147 (PMC10542388; doi:10.7717/peerj.16147)
Supplement: Supplemental Information 7 — Data are shown as the mean (95% confidence interval). Simple main effects are adjusted by Bonferroni correction: P values are multiplied by the number of groups (i.e., 5 for countries and 3 for years). Interaction: P ¡ 0.001, partial η2 = 0.027. [file peerj-11-16147-s007.docx]

Table S6. Human rights restriction by country and year only for the first-time participants. Data are shown as the mean (95% confidence interval). Simple main effects are adjusted by Bonferroni correction: *P* values are multiplied by the number of groups (i.e., 5 for countries and 3 for years). Interaction: *P* < 0.001, partial η^2^ = 0.027.

|  | Japan | The United States | The United Kingdom | Italy | China |
| --- | --- | --- | --- | --- | --- |
| 2020 | 3.71 (3.61–3.81)^e; X^ | 4.07 (3.97–4.17)^d; X^ | 4.95 (4.85–5.05)^b; X^ | 4.47 (4.38–4.56)^c; X^ | 5.95 (5.86–6.03)^a; X^ |
| 2021 | 3.54 (3.41–3.67)^c; X, Y^ | 3.82 (3.69–3.95)^b; Y^ | 3.96 (3.81–4.12)^b; Y^ | 3.95 (3.84–4.06)^b; Y^ | 5.83 (5.73–5.94)^a; X^ |
| 2022 | 3.40 (3.24–3.57)^c; Y^ | 3.44 (3.32–3.56)^c; Z^ | 3.80 (3.66–3.94)^b; Y^ | 3.82 (3.66–3.98)^b; Y^ | 5.84 (5.74–5.94)^a; X^ |

a-e: Different letters represent significant differences (*P* < 0.05) among countries as a simple main effect.

X-Z: Different letters represent a significant difference (*P* < 0.05) among years as a simple main effect.
