# Supplementary material for: Differences in and associations between belief in just deserts and human rights restrictions over a 3-year period in five countries during the COVID-19 pandemic
Source: PeerJ. 2023 Sep 28;11:e16147. doi: 10.7717/peerj.16147 (PMC10542388; doi:10.7717/peerj.16147)
Supplement: Supplemental Information 8 — Data are shown as the mean (95% confidence interval). Simple main effects are adjusted by Bonferroni correction: P values are multiplied by the number of groups (i.e., 5 for countries and 3 for years). BJD is adjusted for different covariates: age (31.5), gender (women = 0.60), academic career (university degree or higher = 0.65), children under junior high school age in the family (presence = 0.29), and elderly people over 65 in the family (presence = 0.23). Interaction: P ¡ 0.001, partial η2 = 0.007. [file peerj-11-16147-s008.docx]

Table S7. Belief in just deserts (BJD) by country and year only for the first-time participants. Data are shown as the mean (95% confidence interval). Simple main effects are adjusted by Bonferroni correction: *P* values are multiplied by the number of groups (i.e., 5 for countries and 3 for years). BJD is adjusted for different covariates: age (31.5), gender (women = 0.60), academic career (university degree or higher = 0.65), children under junior high school age in the family (presence = 0.29), and elderly people over 65 in the family (presence = 0.23). Interaction: *P* < 0.001, partial η^2^ = 0.007.

|  | Japan | The United States | The United Kingdom | Italy | China |
| --- | --- | --- | --- | --- | --- |
| 2020 | 2.31 (2.22–2.39)^a; Y^ | 1.51 (1.43–1.60)^b, c; Y^ | 1.45 (1.37–1.53)^c; Y^ | 1.65 (1.57–1.73)^b; Y^ | 1.67 (1.59–1.75)^b; Y^ |
| 2021 | 2.56 (2.45–2.68)^a; X^ | 1.95 (1.85–2.06)^b; X^ | 1.67 (1.54–1.80)^c; X^ | 1.87 (1.77–1.96)^b, c; X^ | 1.73 (1.64–1.82)^c; Y^ |
| 2022 | 2.74 (2.60–2.88)^a; X^ | 1.88 (1.77–1.98)^b; X^ | 1.57 (1.44–1.69)^c; X, Y^ | 1.72 (1.59–1.85)^b, c; X, Y^ | 1.83 (1.75–1.92)^b; X^ |

a-c: Different letters represent significant differences (*P* < 0.05) among countries as a simple main effect.

X-Y: Different letters represent significant differences (*P* < 0.05) between years as a simple main effect.
