# Supplementary material for: Differences in and associations between belief in just deserts and human rights restrictions over a 3-year period in five countries during the COVID-19 pandemic
Source: PeerJ. 2023 Sep 28;11:e16147. doi: 10.7717/peerj.16147 (PMC10542388; doi:10.7717/peerj.16147)
Supplement: Supplemental Information 9 — Data are shown as the mean (95% confidence interval). Simple main effects are adjusted by Bonferroni correction: P values are multiplied by the number of groups (i.e., 5 for countries and 3 for years). HRR is adjusted for different covariates: age (31.5), gender (women = 0.60), academic career (university degree or higher = 0.65), children under junior high school age in the family (presence = 0.29), and elderly people over 65 in the family (presence = 0.23). Interaction: P ¡ 0.001, partial η2 = 0.027. [file peerj-11-16147-s009.docx]

Table S8. Human rights restriction (HRR) by country and year only for the first-time participants. Data are shown as the mean (95% confidence interval). Simple main effects are adjusted by Bonferroni correction: *P* values are multiplied by the number of groups (i.e., 5 for countries and 3 for years). HRR is adjusted for different covariates: age (31.5), gender (women = 0.60), academic career (university degree or higher = 0.65), children under junior high school age in the family (presence = 0.29), and elderly people over 65 in the family (presence = 0.23). Interaction: *P* < 0.001, partial η^2^ = 0.027.

|  | Japan | The United States | The United Kingdom | Italy | China |
| --- | --- | --- | --- | --- | --- |
| 2020 | 3.68 (3.57–3.78)^e; X^ | 4.06 (3.95–4.16)^d; X^ | 4.92 (4.82–5.02)^b; X^ | 4.46 (4.37–4.55)^c; X^ | 5.99 (5.90–6.09)^a; X^ |
| 2021 | 3.50 (3.37–3.63)^c; X, Y^ | 3.83 (3.70–3.96)^b; Y^ | 3.92 (3.76–4.08)^b; Y^ | 4.00 (3.89–4.12)^b; Y^ | 5.87 (5.76–5.97)^a; X^ |
| 2022 | 3.36 (3.19–3.53)^c; Y^ | 3.43 (3.30–3.55)^c; Z^ | 3.75 (3.61–3.90)^b; Y^ | 3.87 (3.72–4.03)^b; Y^ | 5.88 (5.77–5.98)^a; X^ |

a-e: Different letters represent significant differences (*P* < 0.05) among countries as a simple main effect.

X-Z: Different letters represent a significant difference (*P* < 0.05) among years as a simple main effect.
