# Supplementary material for: Differences in and associations between belief in just deserts and human rights restrictions over a 3-year period in five countries during the COVID-19 pandemic
Source: PeerJ. 2023 Sep 28;11:e16147. doi: 10.7717/peerj.16147 (PMC10542388; doi:10.7717/peerj.16147)
Supplement: Supplemental Information 10 — Controlled variables: age, gender, academic career, and presence or absence of children and elderly people. Bootstrap method with 1000 samples for each analysis is applied to estimate the 95% confidence interval (CI). P values are adjusted by Bonferroni-correction, that is, P values are multiplied by the number of stratified analyses (n = 15). [file peerj-11-16147-s010.docx]

Table S9. Partial correlation between belief in just deserts and human rights restriction by country and year. Controlled variables: age, gender, academic career, and presence or absence of children and elderly people. Bootstrap method with 1000 samples for each analysis is applied to estimate the 95% confidence interval (CI). *P* values are adjusted by Bonferroni-correction, that is, *P* values are multiplied by the number of stratified analyses (n = 15).

|  | Japan | | The United States | | The United Kingdom | | Italy | | China | |
| --- | --- | --- | --- | --- | --- | --- | --- | --- | --- | --- |
|  | *r* (95%CI) | *P* | *r* (95%CI) | *P* | *r* (95%CI) | *P* | *r* (95%CI) | *P* | *r* (95%CI) | *P* |
| 2020 | 0.253 (0.155–0.357) | <0.001 | 0.054 (−0.071–0.173) | 1.000 | −0.025 (−0.154–0.099) | 1.000 | 0.173 (0.080–0.264) | 0.003 | −0.127 (−0.220–−0.040) | 0.063 |
| 2021 | 0.286 (0.194–0.386) | <0.001 | 0.119 (0.018–0.222) | 0.288 | 0.156 (0.043–0.274) | 0.031 | 0.222 (0.112–0.319) | <0.001 | −0.086 (−0.183–0.015) | 1.000 |
| 2022 | 0.468 (0.374–0.558) | <0.001 | 0.145 (0.028–0.255) | 0.065 | 0.040 (−0.081–0.156) | 1.000 | 0.278 (0.169–0.384) | <0.001 | −0.048 (−0.151–0.050) | 1.000 |
