# Supplementary material for: Differences in and associations between belief in just deserts and human rights restrictions over a 3-year period in five countries during the COVID-19 pandemic
Source: PeerJ. 2023 Sep 28;11:e16147. doi: 10.7717/peerj.16147 (PMC10542388; doi:10.7717/peerj.16147)
Supplement: Supplemental Information 11 — Data are shown as the mean (95% confidence interval). Interaction: P = 0.321, partial η2 = 0.008. Min effect: year, P = 0.289, partial η2 = 0.008; country, P < 0.001, partial η2 = 0.131. [file peerj-11-16147-s011.docx]

Table S10. Belief in just deserts by Japan/Italy and by year. Data are shown as the mean (95% confidence interval). Interaction: *P* = 0.321, partial η^2^ = 0.008. Min effect: year, *P* = 0.289, partial η^2^ = 0.008; country, *P* < 0.001, partial η^2^ = 0.131.

|  | Japan | Italy |
| --- | --- | --- |
| 2020 | 2.36 (2.17–2.55) | 1.74 (1.47–2.00) |
| 2021 | 2.42 (2.23–2.61) | 1.92 (1.66–2.18) |
| 2022 | 2.45 (2.26–2.63) | 1.71 (1.45–1.96) |
