# Supplementary material for: Differences in and associations between belief in just deserts and human rights restrictions over a 3-year period in five countries during the COVID-19 pandemic
Source: PeerJ. 2023 Sep 28;11:e16147. doi: 10.7717/peerj.16147 (PMC10542388; doi:10.7717/peerj.16147)
Supplement: Supplemental Information 12 — Data are shown as the mean (95% confidence interval). Interaction: P = 0.084, partial η2 = 0.017. Main effect: year, P < 0.001, partial η2 = 0.183; country, P = 0.034, partial η2 = 0.030. [file peerj-11-16147-s012.docx]

Table S11. Human rights restriction by Japan/Italy and by year. Data are shown as the mean (95% confidence interval). Interaction: *P* = 0.084, partial η^2^ = 0.017. Main effect: year, *P* < 0.001, partial η^2^ = 0.183; country, *P* = 0.034, partial η^2^ = 0.030.

|  | Japan | Italy |
| --- | --- | --- |
| 2020^X^ | 3.96 (3.77–4.15) | 4.19 (3.93–4.45) |
| 2021^Y^ | 3.60 (3.42–3.78) | 4.09 (3.84–4.34) |
| 2022^Z^ | 3.36 (3.16–3.56) | 3.50 (3.22–3.77) |

X-Z: Different letters represent significant differences (*P* < 0.05) among years as the main effect.
