# Supplementary material for: Differences in and associations between belief in just deserts and human rights restrictions over a 3-year period in five countries during the COVID-19 pandemic
Source: PeerJ. 2023 Sep 28;11:e16147. doi: 10.7717/peerj.16147 (PMC10542388; doi:10.7717/peerj.16147)
Supplement: Supplemental Information 13 — P values are adjusted by Bonferroni correction, that is, P values are multiplied by the number of stratified analyses (n = 6). CI: confidence interval. [file peerj-11-16147-s013.docx]

Table S12. Pearson correlation between belief in just deserts and human rights restriction by country and year. *P* values are adjusted by Bonferroni correction, that is, *P* values are multiplied by the number of stratified analyses (n = 6). CI: confidence interval.

|  | Japan |  | Italy |  |
| --- | --- | --- | --- | --- |
|  | *r* (95%CI) | *P* | *r* (95%CI) | *P* |
| 2020 | 0.238 (0.041–0.418) | 0.112 | 0.063 (-0.216–0.333) | 1.000 |
| 2021 | 0.306 (0.113–0.476) | 0.014 | 0.251 (-0.026–0.493) | 0.451 |
| 2022 | 0.323 (0.132–0.491) | 0.008 | 0.594 (0.381–0.748) | <0.001 |
